# Relationships of Dietary Factors with Obesity, Hypertension, and Diabetes by Regional Type among Single-Person Households in Korea

**DOI:** 10.3390/nu13041218

**Published:** 2021-04-07

**Authors:** Kyung Won Lee, Dayeon Shin

**Affiliations:** 1 Department of Home Economics Education, Korea National University of Education, Cheongju 28173, Korea; kwlee@knue.ac.kr; 2 Department of Food and Nutrition, Inha University, Incheon 22212, Korea

**Keywords:** single-person households, regional health disparities, obesity, diabetes, hypertension, Korea Community Health Survey

## Abstract

This study aims to investigate whether dietary factors are differentially associated with metabolic abnormalities by regional type among single-person households in Korea. A total of 685,327 Korean adults aged ≥19 years who participated in the household and individual surveys of the Korea Community Health Survey 2015–2017 are included in the analysis. The regions are divided into three categories: metropolitan areas, mid-sized cities, and rural areas. Using multivariable logistic regressions, the adjusted odds ratios and 95% confidence intervals for metabolic abnormalities are estimated by regional type after adjusting for covariates. Among the total study population, 12.21% occupy single-person households, and 47.5%, 25.1%, and 27.4% of those single-person households are in rural areas, mid-sized cities, and metropolitan areas, respectively. Compared with single-person households in rural areas, those in mid-sized and metropolitan cities tend to be more familiar with and frequently refer to nutrition labels, skip breakfast, and experience food insecurity. Regional differences are found in the associations of dietary factors and behavior with obesity, hypertension, and diabetes. The use of nutritional fact labels is associated with obesity and hypertension in single-person households in rural areas, and the degree of association between food insecurity and diabetes is highest among single-person households in metropolitan areas. Our findings suggest that policies for improving unhealthy dietary factors by regional type are needed to reduce regional health disparities among single-person households in Korea.

## 1. Introduction

As chronic degenerative diseases now occur with greater frequency than infectious diseases, the paradigm of healthcare has shifted from treatment to prevention [1]. Since chronic diseases with a greater socioeconomic burden often stem from poor eating habits and lifestyle patterns, it is important to identify the modifiable risk factors [2]. As part of these efforts, in Korea, the National Health Plan 2020 was launched to achieve the goals of extending a healthy life expectancy and improving health equity. However, health disparities caused by sex, income, and region still exist, and regional disparities in the prevalence of obesity, hypertension, and adherence to a healthy diet have widened [3,4]. In order to eliminate the health disparities among regions, further investigation exploring modifiable factors related to chronic diseases in each regional type is required.

Household types have gradually changed due to the weakening of traditional family values, demonstrated by more individuals choosing to either remain single or form a nuclear family, along with social changes such as improvements in women’s economic activities and an aging population [5,6]. Household type may serve as a key factor that affects aspects of chronic disease occurrence. According to a recent report by Statistics Korea, the rate of single-person households increased almost twofold, from 15.5% in 2000 to 29.8% in 2019, with a single-person household becoming the most common household type in Korea [7,8]. In Korea, single-person households showed the most rapid increase among Organization for Economic Co-operation and Development countries, and the proportion of single-person households is expected to reach 37.7% by 2047 [9].

A few studies have reported the dietary problems that single-person households are facing. Single-person households experience a relative lack of nutritional information and difficulty in achieving a well-balanced diet compared with multi-person households [10]. Lee et al. reported a significant disparity in nutrient intake between single-person households and multi-person households, which has worsened with increasing age and decreasing income [11]. In contrast to multi-person households with two or more family members, single-person households have a low-quality diet and inadequate intake of protein, dietary fiber, calcium, phosphorus, vitamin A, thiamin, riboflavin, niacin, and vitamin C [12]. Furthermore, single-person households spend a greater percentage of their income on food compared with multi-person households. However, they tend to purchase less food and experience difficulty in securing fruits, vegetables, fish, and meat due to the small quantities of foods purchased [11,13].

Previous studies have reported differences in the characteristics and growth patterns of single-person households in each type of region in Korea. A higher proportion of single-person households in Seoul, Daejeon, and Sejong belonged to the younger age group, whereas a large proportion of single-person households in Gangwon-do, Gyeongsang-do, and Jeolla-do belonged to the older age group [14]. Moreover, single-person households with higher education and income levels are prevalent in metropolitan areas, while older single-person households with lower educational levels are prevalent in rural areas [15].

As demonstrated, the characteristics of single-person households vary by region, and individuals living alone are usually confronted with various dietary and nutritional problems. Taking into consideration the abovementioned literature, it is necessary to develop nutritional policies and programs for single-person households by region in order to reduce regional health disparities. However, studies examining the prevalence and modifiable risk factors of chronic diseases in single-person households by regional type are limited. Therefore, here we aim to identify the characteristics of single-person households by regional type and investigate whether the relationships of dietary factors and health-related behavior with obesity, hypertension, and diabetes among single-person households differ by regional type in Korea.

## 2. Materials and Methods

### 2.1. Data Source and Study Population

We used data from the Korea Community Health Survey (KCHS), which is an ongoing nationwide community-based, cross-sectional survey conducted by the Korea Centers for Disease Control and Prevention (KCDC). The KCHS, initiated in 2008, has been conducted annually to gather regional data for planning, implementation, monitoring, and the evaluation of community health services [16]. This survey was designed using complex, stratified, multistage, probability cluster sampling to obtain estimates of non-institutionalized civilian Koreans. The registered population was based on the latest available data from the Ministry of Public Administration and Security. The first stratum was Dong/Eup Myeon (small administrative units) and the second stratum consisted of housing units such as apartments and houses. All household members aged ≥19 years in the sampling point were interviewed. Detailed information on the KCHS has been provided elsewhere [17]. In each survey, a trained interviewer visited the selected households and data on health status, health-related behavior, and diseases were collected through computer-assisted personal interviews. The interview took approximately 20–30 min to complete. All participants provided informed consent and all study protocols and procedures of the KCHS were reviewed and approved by the KCDC Institutional Review Board.

A total of 685,327 participants aged ≥19 years who completed the household and individual surveys as part of the KCHS 2015–2017 were included in this study. We excluded those with missing information on height and weight (*n* = 27,879), diabetes and hypertension diagnoses (*n* = 1074), and sociodemographic, dietary, and health-related characteristics (*n* = 13,697). Among the remaining 642,677 participants, 78,451 (12.21%) belonged to single-person households and 564,226 (87.79%) belonged to multi-person households. Finally, 78,451 single-person households were included in the current analyses.

### 2.2. Definition of Single-Person Households and Regional Types

Participants who responded with “single-person households” to the question “Which of the following best describes your household types?” were classified as single-person households.

The administrative division system of Korea consists of 17 metropolitan cities and provinces: one capital city (Seoul), six metropolitan cities (Busan, Daegu, Incheon, Gwangju, Daejeon, and Ulsan), nine provinces (Gyeonggi-do, Gangwon-do, Chungcheongbuk-do, Chungcheongnam-do, Jeollabuk-do, Jeollanam-do, Gyeongsangbuk-do, Gyeongsangnam-do, and Jeju-do), and one special self-governing city (Sejong). Based on the administrative division system in Korea, regional type is divided into metropolitan regions (Seoul and six metropolitan cities), mid-sized cities (“*Dong*” areas of nine provinces and Sejong), and rural areas (“*Eup*” and “*Myeon*” areas of nine provinces and Sejong).

### 2.3. Dietary and Health-Related Characteristics

Data on dietary and health-related behaviors were collected by a trained interviewer via one-on-one interviews. To assess the dietary and health-related characteristics of single-person households, data on breakfast skipping, adherence to low-sodium diets, recognition and use of nutritional fact labels, household food insecurity, and adherence to healthy lifestyle practices were obtained. Breakfast skipping was defined as taking less than five breakfasts in a week. Adherence to low-sodium diets was defined as usually consuming bland diet and not adding salt or soy sauce at all when eating cooked food. With regards to the recognition and use of nutritional fact labels, the participants were classified into two groups (yes or no) by asking the following questions: “Do you know about nutritional fact labels?” and “Do you read nutritional fact labels when you purchase processed foods?”. Household food insecurity was assessed using a one-item food insecurity questionnaire. Participants who answered “there was not enough to eat sometimes/often due to financial difficulties” were classified as having household food insecurity. Adherence to healthy lifestyle practices was defined as non-smoking persons (non-smokers or past smokers), engaging in moderate drinking (non-drinkers or individuals who consumed seven or fewer drinks in men or five or fewer drinks in women on one occasion a week) and regular walking (walking ≥30 min ≥5 days a week; walking time includes leisure-time walking and walking to school, work, or for shopping).

### 2.4. Definition of Metabolic Abnormalities: Obesity, Diabetes, and Hypertension

Body mass index (BMI) was calculated as weight (kg) divided by height in meters squared (m^2^). Obesity was defined as a BMI of ≥25 kg/m^2^ based on the Asia-Pacific region of the World Health Organization’s criteria [18]. For diabetes and hypertension, KCHS participants were asked the following question: “Has a doctor ever told you that you have diabetes/hypertension?”. Participants who responded “yes” to the question were classified as having diabetes or hypertension.

### 2.5. Statistical Analyses

All statistical analyses were conducted using SAS (version 9.4, SAS Institute Inc., Cary, NC, USA). A two-sided *p*-value of <0.05 was considered significant. To account for the complex sampling design of the KCHS, the PROC SURVEY procedure with the sample weight, stratum, and primary sampling unit was used, as recommended by the KCDC analytic guidelines [16].

The sociodemographic characteristics and dietary and health-related behaviors of single-person households according to regional type were analyzed using the chi-square test for categorical variables and linear regression analysis for continuous variables. We conducted multiple logistic regression analyses to determine associations of regional type with the odds of obesity, hypertension, and diabetes stratified by sex and age group. The adjusted odds ratios (AORs) and 95% confidence intervals (CIs) for obesity, hypertension, and diabetes according to dietary and health-related characteristics and unhealthy behaviors by regional type among single-person households were also estimated. Potential covariates including sex (man or woman), age (continuous), education level (≤middle school, high school, and ≥ college), household income (<100, 100 to <200, 200 to <300, and ≥300 Korean won/month), marital status (single or married), occupation (white-collar, blue-collar, or unemployed), current drinking (yes or no), current smoking (yes or no), and regular physical activity (performing vigorous activity ≥3 times a week for ≥20 min each time or moderate activity ≥5 times a week for ≥30 min each time; yes or no) were adjusted.

## 3. Results

Of the total of 642,677 participants from the KCHS 2015–2017, 12.21% (*n* = 78,451) were single-person households. Among individuals living alone, 47.48% (*n* = 38,357), 25.09% (*n* = 19,026), and 27.43% (*n* = 21,068) lived in rural areas, mid-sized cities, and metropolitan areas, respectively. The general characteristics of single-person households according to regional types are listed in Table 1. Single-person households in rural areas tended to constitute women, older people, unemployed individuals, and those with a lower household income level (all, *p* < 0.0001). Individuals in single-person households in rural areas were less likely to be highly educated, single, current smokers, drinkers, or obese, and less likely to perform regular physical activity or adhere to healthy lifestyle practices (all, *p* < 0.05).

The multivariable-AORs for obesity, hypertension, and diabetes according to regional type in single-person households are presented in Table 2. After controlling for covariates including sex, age, education and household income level, occupation, current drinking and smoking status, and physical activity, single-person household members in mid-sized cities showed higher odds of obesity (AOR: 1.14, 95% CI: 1.09–1.19) and diabetes (AOR: 1.16, 95% CI: 1.09–1.22) than those living in rural areas. Compared with single-person household members in rural areas, those living in metropolitan areas were more likely to have diabetes (AOR: 1.14, 95% CI: 1.08–1.21). When stratified by sex, men in single-person households in mid-sized cities had higher odds of hypertension (AOR: 1.10, 95% CI: 1.02–1.18) and those living in metropolitan areas had higher odds of diabetes (AOR: 1.15, 95% CI: 1.05–1.26) and hypertension (AOR: 1.08, 95% CI: 1.01–1.16). Women in single-person households in mid-sized cities and metropolitan areas had higher odds of obesity and diabetes compared with those living in rural areas. When stratified by age, individuals aged 19–39 years old in single-person households in mid-sized cities and metropolitan areas showed lower odds of obesity (mid-sized city residents = AOR: 0.89, 95% CI: 0.80–0.98; metropolitan area residents = AOR: 0.77, 95% CI: 0.69–0.85). Among individuals aged 40–64 years old in single-person households, those living in metropolitan areas were less likely to be obese (AOR: 0.88, 95% CI: 0.82–0.94) or have hypertension (AOR: 0.93, 95% CI: 0.87–0.99) compared with those living in rural areas. In adults aged ≥65 years old living alone, the odds of obesity and diabetes increased, but the odds of hypertension decreased for individuals living in mid-sized cities and metropolitan areas, compared with those living in rural areas.

The dietary and health-related characteristics of single-person households according to regional type are shown in Table 3. Overall, compared with single-person households in rural areas, those living in mid-sized cities and metropolitan areas were more likely to adhere to healthy lifestyle practices (mid-sized city residents = AOR: 1.24, 95% CI: 1.19–1.30; metropolitan area residents = AOR: 1.70, 95% CI: 1.63–1.77), recognize nutritional fact labels (mid-sized city residents = AOR: 1.15, 95% CI: 1.10–1.21; metropolitan area residents = AOR: 1.09, 95% CI: 1.04–1.15), and use nutritional fact labels (mid-sized city residents = AOR: 1.14, 95% CI: 1.08–1.21; metropolitan area residents = AOR: 1.18, 95% CI: 1.11–1.25). They also tended to experience food insecurity (mid-sized city residents = AOR: 1.42, 95% CI: 1.33–1.52; metropolitan area residents = AOR: 1.65, 95% CI: 1.55–1.76), but were less likely to skip breakfast (mid-sized city residents = AOR: 0.77, 95% CI: 0.73–0.81; metropolitan area residents = AOR: 0.78, 95% CI: 0.74–0.82).

The relationships of unhealthy dietary factors with obesity, hypertension, and diabetes among single-person households according to regional types are shown in Table 4. A significant association between non-adherence to low-sodium diets and obesity was found in all regional types. Not using nutritional fact labels was positively associated with obesity in individuals living alone in rural areas (AOR: 1.17, 95% CI: 1.07–1.28). Non-adherence to healthy living practices was positively associated with obesity in single-person household members in mid-sized cities (AOR: 1.10, 95% CI: 1.02–1.18) and metropolitan areas (AOR: 1.10, 95% CI: 1.02–1.17). In addition, breakfast skipping was associated with increased odds of obesity in mid-sized cities (AOR: 1.12, 95% CI: 1.03–1.21). In terms of diabetes, breakfast skipping, non-adherence to low-sodium diet practices, and food insecurity were positively associated with diabetes among single-person households living in all three regional types. Rural residents not adhering to healthy lifestyle practices had higher odds of diabetes (AOR: 1.09, 95% CI: 1.03–1.17) than those adhering to healthy lifestyle practices. Not recognizing nutritional fact labels was associated with increased odds of diabetes among rural (AOR: 1.09, 95% CI: 1.01–1.19) and metropolitan residents (AOR: 1.15, 95% CI: 1.03–1.28). The odds of hypertension increased among rural residents with breakfast skipping, non-adherence to low-sodium diets, and not using nutritional fact labels. Mid-sized city residents showing non-adherence to healthy lifestyle practices and metropolitan residents who skipped breakfast had higher odds of hypertension.

## 4. Discussion

In this cross-sectional study based on a nationwide representative sample of the Korean population, approximately 12% of the participants lived in a single-person household, of whom 47%, 25%, and 27% lived in rural areas, mid-sized cities, and metropolitan areas, respectively. The characteristics of single-person households differed significantly according to the regional type; individuals in single-person households in rural areas were more likely to be older and with lower education and household income, while those living in metropolitan areas were younger and had higher education and income levels. This finding is concordant with that of previous studies, which reported single-person households with higher education and income levels to be more commonly found in the capital and metropolitan cities, while older people living alone with lower education levels usually reside in rural areas [14,15].

We found that unhealthy dietary factors were differentially associated with chronic diseases among single-person households, depending on the type of region. Adherence to low-sodium diets and breakfast skipping were among the dietary factors associated with metabolic abnormalities, suggesting regional differences in risk estimates. This result is in line with that of previous studies, which reported that consumption of food with lower dietary salt helps to lower blood pressure and reduces the risk of cardiovascular diseases. In addition, reducing salt by 1 g a day may decrease medical expenses due to hypertension [19]. In another analysis of the KCHS 2008, current drinkers and those who drank more frequently were found to be less likely to consume low-sodium diets [20]. This finding indicated that adherence to a low-sodium diet might have been associated with other healthy lifestyles. Breakfast skipping is one of the dietary behaviors that needs to be improved in single-person households. Previous studies have demonstrated that single-person households are more likely to skip breakfast and have irregular meal patterns than multi-person households [11,21]. In particular, skipping breakfast has been significantly related to obesity, excess weight, and poor dietary quality [22]; thus, effective strategies for promoting low-sodium diet practices and breakfast consumption should be considered when designing nutritional interventions for single-person households.

The association of nutritional fact label use with obesity, hypertension, and diabetes is evident among single-person households in rural areas. The use of nutritional fact labels has been reported to be significantly related to reduced BMI and lower odds of obesity and diabetes in diverse populations [23,24,25]. Moreover, the use of nutritional fact labels in the management of chronic diseases is helpful [26] and has been emphasized in nutritional education, in order to maintain a good nutritional status and health. The results of this study indicate that the proportion of single-person households in rural areas who did not use nutritional fact labels was higher than those living in mid-sized and metropolitan cities. This finding suggests the importance of education on nutrition label use for single-person households in rural areas.

Interestingly, food insecurity was significantly associated with increased odds of diabetes among single-person households, regardless of regional type. In addition, higher AORs of food insecurity were observed in single-person households in mid-sized and metropolitan cities, compared with those in rural areas. According to data from the Korea National Health and Nutrition Examination Survey 2013, single-person households were twice as likely to experience food insecurity compared with households with more than two people (18% vs. 9%) [11]. Previous studies have reported that income or employment status is significantly related to food insecurity; in particular, the odds of food insecurity increase among individuals with lower income [27] or who are unemployed [28,29]. Individuals in single-person households tend to have higher employment and income insecurity, and they often do not receive tax benefits [30]; moreover, single-person households frequently face financial difficulties when compared with multi-person households. This implies the need to establish nutrition programs or policies that can ensure the food security of single-person households.

To the best of our knowledge, this is the first study to fill important gaps in existing knowledge regarding the regional health disparities of single-person households. In addition, the strengths of the present study include the use of data from a large, representative sample of Korean adults and adjustment for various covariates that may affect both dietary behaviors and the prevalence of obesity, hypertension, and diabetes. However, the present study has some limitations. First, due to the cross-sectional nature of the study, it is difficult to establish causal relationships between unhealthy dietary behaviors and metabolic abnormalities. Second, the KCHS obtained data on some dietary behaviors such as breakfast skipping, adherence to low-sodium diets, and nutritional fact label usage, which were included in the present study; however, data regarding the participants’ actual dietary intake was not collected. The lack of information on dietary intake led to some limitations in drawing conclusions regarding the association between dietary intake and metabolic abnormalities. Third, the KCHS data were based on self-reported information, which may cause recall bias and systematic reporting errors. Furthermore, the prevalence of obesity, hypertension, and diabetes in this study was defined based on self-reported physicians’ diagnoses of diabetes or hypertension. Lifetime physicians’ diagnoses of diseases generally indicate an individual’s recognition of having certain diseases. However, since the objective of this study was to identify the characteristics and dietary factors related to metabolic abnormalities in single-person households by regional type, proportions of self-reported lifetime physician-diagnosed obesity, hypertension, and diabetes were considered as a prevalence [4,31]. Further investigation should be conducted using nationally representative samples with actual dietary intake and laboratory data that can accurately determine an individual’s nutritional status and the presence of chronic diseases. As the era of six million single-person households arrives, single-person households are rising as a central player in social and economic changes that lead to new consumer trends. Single-person households are becoming increasingly common and are expected to account for about 40 percent of the total households in Korea. Continuous efforts to identify the chronic disease-related dietary and lifestyle risk factors of single-person households by regional type will help to overcome worsening regional health disparities.

## 5. Conclusions

In summary, our findings demonstrate that the associations of unhealthy dietary factors and behaviors with obesity, hypertension, and diabetes among single-person households differ by regional type. Skipping breakfast is associated with metabolic abnormalities among single-person households in all three types of regions. In rural areas, the recognition and use of nutritional fact labels are significantly associated with metabolic abnormalities, while the strongest associations between household food insecurity and metabolic abnormalities are observed among those living in metropolitan areas. Recently, nutritional education has been provided at the local level to address the nutritional problems faced by single-person households, such as irregular meal patterns and nutritional imbalances. However, single-person households remain unaware of different health and nutritional policies at a national level. Therefore, further regional strategies for improving unhealthy dietary factors are needed to reduce health disparities by region, particularly in light of a growing population of single-person households in Korea.

## Figures and Tables

**Table 1:** General characteristics of single-person households by regional type in Korea: KCHS 2015–2017 ^1^.

	Total	Regional Type			*p*-Value ^2^
		Rural Areas	Mid-Sized Cities	Metropolitan Areas	
	*n* (Wt’d %)	*n* (Wt’d %)	*n* (Wt’d %)	*n* (Wt’d %)	
Total	78,451 (100.00)	38,357 (47.48)	19,026 (25.09)	21,068 (27.43)	
Sex					<0.0001
Men	28,942 (42.17)	12,437 (37.25)	8085 (47.93)	8420 (45.41)	
Women	49,509 (57.83)	25,920 (62.75)	10,941 (52.07)	12,648 (54.59)	
Age (years)	59.24 ± 0.08 ^3^	65.31 ± 0.10	53.60 ± 0.16	53.92 ± 0.15	<0.0001
Education level					<0.0001
≤Middle school	33,930 (39.81)	22,965 (55.94)	5313 (25.71)	5652 (24.79)	
High school	25,954 (34.33)	10,471 (29.42)	7635 (40.24)	7848 (37.42)	
≥College	18,567 (25.86)	4921 (14.64)	6078 (34.05)	7568 (37.80)	
Household income (10,000 KRW/month)					<0.0001
<100	44,709 (54.05)	26,570 (66.11)	8324 (41.51)	9815 (44.66)	
100 to <200	15,728 (20.55)	6106 (16.92)	4581 (23.76)	5041 (23.88)	
200 to <300	10,306 (14.41)	3239 (9.58)	3531 (19.85)	3536 (17.78)	
≥300	7708 (11.00)	2442 (7.39)	2590 (14.88)	2676 (13.68)	
Marital status					<0.0001
Single	71,765 (90.82)	34,848 (89.79)	17,493 (91.58)	19,424 (91.91)	
Married	6686 (9.18)	3509 (10.21)	1533 (8.42)	1644 (8.09)	
Occupation					<0.0001
White-collar	10,671 (14.71)	2668 (7.85)	3666 (20.29)	4337 (21.47)	
Blue-collar	31,456 (41.13)	17,077 (45.85)	7305 (39.36)	7074 (34.57)	
Unemployed	36,324 (44.16)	18,612 (46.30)	8055 (40.34)	9657 (43.96)	
BMI status					<0.0001
Underweight	1246 (4.36)	2876 (5.48)	952 (3.62)	1246 (4.36)	
Normal weight	9466 (40.73)	17,230 (40.82)	8255 (38.96)	9466 (40.73)	
Overweight	5108 (25.20)	9201 (25.30)	4614 (24.85)	5108 (25.20)	
Obesity	5248 (29.71)	9050 (28.40)	5205 (32.57)	5248 (29.71)	
Current drinking					<0.0001
No	44,498 (53.88)	24,776 (61.81)	9254 (46.11)	10,468 (47.28)	
Yes	33,953 (46.12)	13,581 (38.19)	9772 (53.89)	10,600 (52.72)	
Current smoking					<0.0001
No	62,371 (77.31)	31,961 (81.35)	14,166 (72.29)	16,244 (74.90)	
Yes	16,080 (22.69)	6396 (18.65)	4860 (27.71)	4824 (25.10)	
Physical activity ^4^					0.0152
Yes	15,122 (20.14)	7298 (19.79)	3792 (20.91)	4032 (20.06)	
No	63,329 (79.86)	31,059 (80.21)	15,234 (79.09)	17,036 (79.94)	
Adherence to healthy lifestyle practices ^5^					<0.0001
Yes	21,895 (27.28)	9402 (24.09)	5197 (26.45)	7296 (33.56)	
No	56,556 (72.72)	28,955 (75.91)	13,829 (73.55)	13,772 (66.44)	

^1^ All data were weighted to account for the complex study design according to the KCHS analytical guidelines. ^2^ *p*-values were obtained from the Wald chi-square test. ^3^ Mean standard ± error (all such variables). ^4^ Physical activity was defined as vigorous activity ≥3 times a week for ≥20 min each time or moderate activity ≥5 times a week for ≥30 min each time. ^5^ Adherence to healthy lifestyle practices was defined as moderate drinking, non-smoking, and regular walking. KCHS, Korea Community Health Survey; Wt’d %, weighted percentage; KRW, Korean won.

**Table 2:** Adjusted odds ratios with 95% confidence intervals for obesity, hypertension, and diabetes by regional type among single-person households in Korea: KCHS 2015–2017 ^1^.

	Regional Type		
	Rural Areas	Mid-Sized Cities	Metropolitan Areas
		AOR (95% CI)	AOR (95% CI)
Total			
Obesity	1.00 (ref.)	1.14 (1.09–1.19) ^2^	1.02 (0.98–1.07)
Diabetes	1.00 (ref.)	1.16 (1.09–1.22)	1.14 (1.08–1.21)
Hypertension	1.00 (ref.)	1.03 (0.99–1.08)	0.96 (0.92–1.00)
Men			
Obesity	1.00 (ref.)	1.05 (0.98–1.12)	0.95 (0.89–1.01)
Diabetes	1.00 (ref.)	1.09 (0.99–1.20)	1.15 (1.05–1.26)
Hypertension	1.00 (ref.)	1.10 (1.02–1.18)	1.08 (1.01–1.16)
Women			
Obesity	1.00 (ref.)	1.22 (1.15–1.29)	1.12 (1.05–1.18)
Diabetes	1.00 (ref.)	1.21 (1.13–1.29)	1.15 (1.08–1.23)
Hypertension	1.00 (ref.)	1.01 (0.95–1.06)	0.91 (0.86–0.96)
19–39 years			
Obesity	1.00 (ref.)	0.89 (0.80–0.98)	0.77 (0.69–0.85)
Diabetes	1.00 (ref.)	0.98 (0.62–1.55)	0.90 (0.56–1.47)
Hypertension	1.00 (ref.)	1.09 (0.85–1.38)	1.07 (0.84–1.37)
40–64 years			
Obesity	1.00 (ref.)	0.96 (0.90–1.02)	0.88 (0.82–0.94)
Diabetes	1.00 (ref.)	0.95 (0.86–1.05)	0.97 (0.88–1.06)
Hypertension	1.00 (ref.)	0.95 (0.89–1.02)	0.93 (0.87–0.99)
65+ years			
Obesity	1.00 (ref.)	1.49 (1.40–1.59)	1.33 (1.25–1.41)
Diabetes	1.00 (ref.)	1.25 (1.17–1.33)	1.21 (1.14–1.29)
Hypertension	1.00 (ref.)	0.99 (0.93–1.04)	0.90 (0.85–0.95)

^1^ All data were weighted to account for the complex study design according to the KCHS analytical guidelines. ^2^ Adjusted for sex, age, education level, household income, marital status, occupation, current drinking, current smoking, and regular physical activity. KCHS, Korea Community Health Survey; AOR, adjusted odds ratio; CI, confidence interval.

**Table 3:** Adjusted odds ratios with 95% confidence intervals for dietary and health-related characteristics by regional type among single-person households in Korea: KCHS 2015–2017 ^1^.

	Regional Type		
	Rural Areas	Mid-Sized Cities	Metropolitan Areas
		AOR (95% CI)	AOR (95% CI)
Breakfast skipping	1.00 (ref.)	0.77 (0.73–0.81) ^2^	0.78 (0.74–0.82)
Adherence to low-sodium diets	1.00 (ref.)	1.01 (0.95–1.06)	0.98 (0.93–1.04)
Recognition of nutritional fact labels	1.00 (ref.)	1.15 (1.10–1.21)	1.09 (1.04–1.15)
Using nutritional fact labels	1.00 (ref.)	1.14 (1.08–1.21)	1.18 (1.11–1.25)
Food insecurity	1.00 (ref.)	1.42 (1.33–1.52)	1.65 (1.55–1.76)
Adherence to healthy lifestyle practices	1.00 (ref.)	1.24 (1.19–1.30)	1.70 (1.63–1.77)

^1^ All data were weighted to account for the complex study design according to the KCHS analytical guidelines. ^2^ Adjusted for sex, age, education level, household income, marital status, occupation, current drinking, current smoking, and regular physical activity. KCHS, Korea Community Health Survey; AOR, adjusted odds ratio; CI, confidence interval.

**Table 4:** Associations of unhealthy behaviors and dietary factors with obesity, hypertension, and diabetes by regional type among single-person households in Korea: KCHS 2015–2017 ^1^.

	Regional Type		
	Rural Areas	Mid-Sized Cities	Metropolitan Areas
	AOR (95% CI)	AOR (95% CI)	AOR (95% CI)
Obesity			
Breakfast skipping	0.98 (0.91–1.05) ^2^	1.12 (1.03–1.21)	1.07 (0.99–1.16)
Non-adherence to low-sodium diets	1.08 (1.01–1.16)	1.13 (1.02–1.24)	1.22 (1.11–1.35)
No recognition of nutritional fact labels	0.99 (0.93–1.05)	0.95 (0.88–1.02)	0.98 (0.91–1.05)
Not using nutritional fact labels	1.17 (1.07–1.28)	0.96 (0.87–1.05)	1.07 (0.98–1.17)
Food insecurity	0.94 (0.87–1.02)	0.94 (0.84–1.05)	1.03 (0.93–1.14)
Non-adherence to healthy lifestyle practices	1.03 (0.98–1.10)	1.10 (1.02–1.18)	1.10 (1.02–1.17)
Diabetes			
Breakfast skipping	1.32 (1.19–1.47)	1.31 (1.14–1.50)	1.28 (1.13–1.46)
Non-adherence to low-sodium diets	1.30 (1.21–1.40)	1.19 (1.06–1.35)	1.16 (1.03–1.30)
No recognition of nutritional fact labels	1.09 (1.01–1.19)	1.02 (0.91–1.13)	1.15 (1.03–1.28)
Not using nutritional fact labels	1.13 (0.99–1.28)	1.15 (0.98–1.36)	1.17 (1.00–1.36)
Food insecurity	1.18 (1.09–1.28)	1.14 (1.00–1.29)	1.24 (1.10–1.39)
Non-adherence to healthy lifestyle practices	1.09 (1.03–1.17)	0.97 (0.87–1.07)	0.97 (0.88–1.06)
Hypertension			
Breakfast skipping	1.15 (1.06–1.24)	0.99 (0.90–1.09)	1.10 (1.00–1.21)
Non-adherence to low-sodium diets	1.14 (1.07–1.22)	1.02 (0.92–1.12)	1.06 (0.96–1.17)
No recognition of nutritional fact labels	1.02 (0.98–1.07)	1.02 (0.96–1.09)	0.97 (0.89–1.06)
Not using nutritional fact labels	1.13 (1.03–1.24)	1.10 (0.98–1.23)	1.06 (0.95–1.18)
Food insecurity	0.99 (0.93–1.06)	1.04 (0.93–1.16)	1.10 (1.00–1.22)
Non-adherence to healthy lifestyle practices	0.98 (0.94–1.04)	1.11 (1.02–1.20)	1.06 (0.99–1.14)

^1^ All data were weighted to account for the complex study design according to the KCHS analytical guidelines. ^2^ Adjusted for sex, age, education level, household income, marital status, occupation, current drinking, current smoking, and regular physical activity. KCHS, Korea Community Health Survey; AOR, adjusted odds ratio; CI, confidence interval.

## Data Availability

The KCHS data used in the manuscript can be found at the following link: https://chs.cdc.go.kr/chs/rdr/rdrInfoDownMain.do (accessed on 1 May 2020).
